# The effect of autochthonous caproic acid-producing consortia inoculation on the multi-dimensional prokaryotic succession of bottom pitmud used for the Chinese strong-flavor Baijiu fermentation

**DOI:** 10.1016/j.crmicr.2025.100490

**Published:** 2025-10-14

**Authors:** Huimin Zhang, Li Zhen, Qiang Chang, Yan Liu, Kangjie Xu, Xiuben Wang, Lei Cui, Zaijie Wu, Zhenglian Xue

**Affiliations:** a Engineering Laboratory for Industrial Microbiology Molecular Beeding of Anhui Province, College of Biologic &Food Engineering, Anhui Polytechnic University, 8 Middle Beijing Road, Wuhu 241000, China; b Anhui Wenwang Distillery Co., Ltd, Linquan city 236400, Anhui Province, China

**Keywords:** Pitmud aging, CPBs-rich consortia, Prokaryotic community, Physicochemical property, “Lag-aging dual-phase” hypothesis

## Abstract

Caproic acid-producing bacteria (CPBs) are considered of great importance to the aging of pitmud within the Chinese strong-flavor Baijiu ecosystem. However, the role of CPBs during pitmud aging remains unclear. To address this question, autochthonous CPBs were subjected to successive liquid-state enrichment and solid-state pre-cultivation with fresh bottom pitmud, followed by five consecutive batches of fermentation to artificially accelerate pitmud aging. This study revealed how CPBs and related prokaryotic communities evolved during these stages. The enriched CPBs did not grow as rapidly in the solid-state new pitmud as in the liquid state. The multi-dimensional succession pattern of bottom pitmud prokaryotic communities, α-diversities, β-diversities, physicochemical properties, and the predicted caproic acid synthesis functions co-verified the “Lag-aging dual-phase” hypothesis proposed in this study. This hypothesis divides pitmud aging into two distinct phases: a “Lag phase” (adaptive phase) and an “aging phase”. After five fermentation batches, the prokaryotic community structure of the new bottom pitmud closely resembled that of aged old bottom pitmud, and the CPBs gradually concentrated in the surface layer (0–1 cm). This study therefore confirmed that the inoculation with autochthonous CPBs-rich consortia facilitated pitmud aging, paving the way for artificial pitmud aging and targeted bioremediation.

## Highlights

• The CPBs-rich consortia could be enriched from huangshui-pitmud co-fermentation.
• The inoculation of autochthonous CPBs-rich consortia promotes bottom pitmud aging.
• The pitmud aging involved two distinct phases: the “Lag phase” and “Aging phase”.

## Graphical abstract

**Figure:**
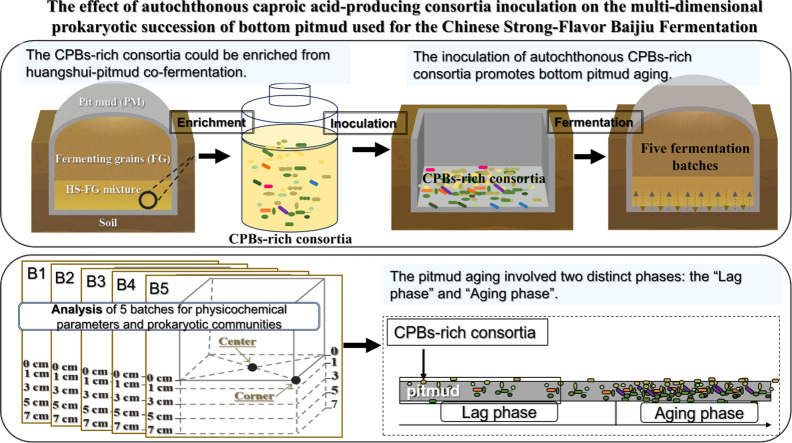

## 1 Introduction

**Fig. 1:**
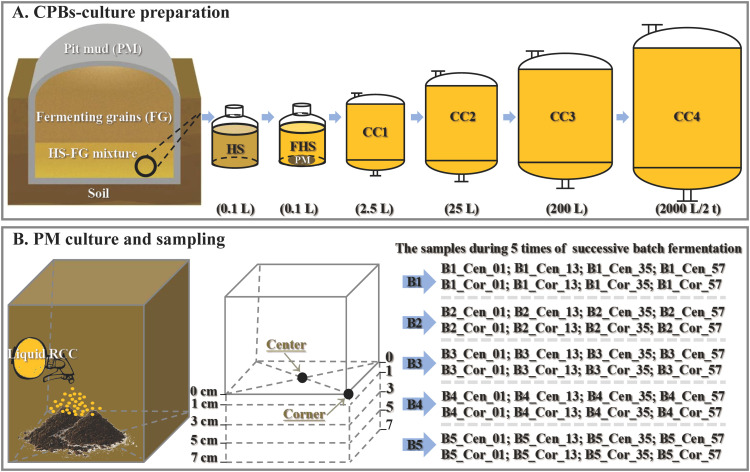
The procedure of the preparation of liquid caproic acid-producing bacteria (CPBs) culture (A), the pitmud pre-culturing and the multi-site pitmud sampling after 5 times of successive batch-fermentation (B).

Traditional Chinese strong-flavor Baijiu (CSFB) is produced by batch fermentation in underground rectangular pits (like simple fermenters) (Fig.1A). The multi-dimensional bottom pit-mud (hereinafter referred to as the technical term ‘pitmud’) is the main source of prokaryotic anaerobes (Wang et al., 2017; Zhang et al., 2020; Gao et al., 2021a). In the later stage of each batch of solid-state CSFB fermentation, the percolate of fermenting grains – huangshui infiltrates to deposit at the bottom of the pit (Ren et al., 2024), which form the solid-liquid interface where pitmud and huangshui interact – pitmud-huangshui co-fermentation microenvironment. Meanwhile, liquid-phase huangshui promotes the production of components that enhance flavor, such as caproic acid (Gao et al., 2020, 2022a; Zhou et al., 2023a). During long-term batch fermentations, liquid-phase huangshui provides potential sources of carbon (e.g. lactic acid, ethanol, reducing sugar, and glucose) and nitrogen (NH_4_^+^-N), as well as other nutrients, which promote the growth of anaerobes, including caproic acid-producing bacteria (CPBs), in pitmud (Kang et al., 2022; Gao et al., 2023; Ren et al., 2024; Zhang et al., 2024). As pitmud ages, both the relative and absolute abundances of CPBs increase (Hu et al., 2016; Zhang et al., 2020). Accordingly, higher levels of caproic acid are produced by aged (mature) pitmud. Caproic acid is acknowledged to be one of the key flavor components of CSFB (Wang et al., 2022a; Hong et al., 2023; Chen et al., 2024a), as described in the national standard GB/T 10,781.1–2021 “Liquor Quality Requirements Part 1: Strong-Flavor Baijiu”. Also, CPBs-rich consortia have been proven to promote the production of caproic acid during CSFB fermentation (Gao et al., 2022a).

Since the 1960s, significant progress has been made to artificially enhance the quantity of CPBs in pitmud (Ding et al., 2014). Pitmud is traditionally produced using a mixture of used pitmud, clay, humus, fermented grain, huangshui, Daqu (fermentation starter), tail Baijiu (obtained from the final stage of distillation) (Ding et al., 2014; Zhang et al., 2015; Liu et al., 2022). Most related studies have mainly focused on the structure and function of the microbial community of pitmud (Ding et al., 2014; Zhang et al., 2015; Liu et al., 2020; Fu et al., 2021; Hu et al., 2016; Zhang et al., 2020; Liu et al., 2022). Prior studies of pitmud primarily employed a single-dimensional surface sampling method. After deciphering the core mechanisms underlying caproic acid- synthesis in pitmud, subsequent research has focused on methods to improve proliferation of CPBs in pitmud (Liu et al., 2020; Gao et al., 2021a, 2021b; Qian et al., 2021; Gao et al., 2022a; Yan et al., 2023; Zhou et al., 2024). CPBs with various other species in pitmud synergistically synthesize caproic acid (Qian et al., 2021; Tu et al., 2022; Kang et al., 2024; Gao et al., 2022b; Yuan et al., 2022). Further studies confirmed that multiple rounds of liquid enrichment using a suitable medium (e.g. Clostridial growth medium) successfully enriched autochthonous CPBs, *Clostridium*, and/or *Caproicibacterium* from pitmud (Zhu et al., 2015; Wang et al., 2021; Li et al., 2023). Our group previously reported that co-fermentation of pitmud and huanghui under suitable physicochemical conditions promoted the proliferation of *Caproicibacterium* and *Clostridium* (Zhang et al., 2025). At the genus level, the most abundant CPBs isolated from pitmud mainly include *Clostridium* (Liu et al., 2014; Wang et al., 2018; Xu et al., 2019), *Caproicibacterium* (Zhu et al., 2015; Wang et al., 2021; Gu et al., 2021, 2022b; Zeng et al., 2024)*,* and *Caproicibacter* (Dai et al., 2024). Studies of pure liquid culture have demonstrated that CPBs isolated from pitmud can use glucose, ethanol, or lactic acid as carbon sources to produce caproic acid via the reverse β-oxidation (RBO) and fatty acid biosynthesis (FAB) pathways (Fig. S1) (Dong et al., 2023; Liu et al., 2024). Recent studies have explored the impact of both CPBs-rich consortia and *Caproicibacterium lactatifermentans* monoculture on the prokaryotic community structure of pitmud (Liu et al., 2020, 2022; Zhou et al., 2024). However, studies of single-dimensional surface pitmud samples found that artificial inoculation of CPBs into pitmud could only increase the short-term abundance of *Clostridium* or *Caproicbacterium*, while long-term fermentation reduced the proportion of *Caproicbacterium*, which was inconsistent with the known accumulation of CPBs in aged pitmud (Tao et al., 2014; Hu et al., 2016; Zhang et al., 2020). Moreover, no substantial production of caproic acid has been observed to date in CPBs-rich solid-state pitmud. Therefore, we hypothesize that there may be differences in the metabolic patterns of CPBs in complex multiphase solid-state pitmud systems versus homogeneous liquid systems (Zhang et al., 2017; Zhang et al., 2020). The succession pattern of CPBs in the multi-dimensional prokaryotic community of bottom pitmud has not yet been investigated. Hence, in-depth investigations are needed to clarify differences in the growth and metabolic characteristics of CPBs in liquid-state versus solid-state cultures, and the multi-dimensional succession patterns following artificial inoculation with autochthonous CPBs.

Therefore, this paper aimed to: (1) obtain autochthonous CPBs-rich consortia from pitmud-huangshui liquid co-fermentation based on our previous study (Zhang et al., 2025); (2) pre-culture the CPBs-rich consortia together with solid new pitmud to preliminarily reveal the single-dimensional difference between liquid-state and solid-state growth status of CPBs; (3) further explore the multi-dimensional succession patterns of prokaryotic communities (growth) and the associated physicochemical properties (metabolism) during five consecutive batches of CSFB fermentation, verifying whether the inoculated CPBs could promote the aging of bottom pitmud. Of note, the fermentation quality of CSFB involves additional variables influencing the fermentation process, which will be investigated in future studies.

## 2 Materials and methods

### 2.1 Huangshui, bottom pitmud sampling, and enrichment of CPBs

Fresh huangshui and bottom pitmud samples were collected from 3 normally used old pits (≥ 40 years old) of a CSFB workshop located in northern Anhui province, China. The huangshui samples were collected via pre-embedded pipes along the pit walls after 40 days of fermentation (total fermentation period: 60 days). The bottom pitmud samples were collected from the surface-layer 0–1 cm. The above huangshui and pitmud samples were each equally mixed well and temporarily stored in sterile anaerobic bottles and sterile bags, respectively, at 4 °C for later enrichment of CPBs.

Based on our earlier research (Zhang et al., 2025), the mixture (1:10) of above pitmud and pre-diluted huangshui (1:1 mixed with deionized water), with an adjusted pH of 6.5, was anaerobically co-fermented in triplicate for enrichment of CPBs. Additional glucose (10 g/L) was added into the above system to produce a near natural huangshui (NNHS) medium without sterilization (Table S2). The surface layer (depth: 0–1 cm) of old-pit bottom pitmud is known to be rich in CPBs (Zhang et al., 2020; Zhao et al., 2024). So, the surface bottom pitmud inoculated into unsterilized NNHS medium primarily served as the CPBs source. And, huangshui served as sources of both CPBs and nutrient. Triplicates of the above 70 mL system were loaded into 100 mL anaerobic bottles and placed in an anaerobic workstation (Don Whitley A20, UK) filled with anaerobic gases (80 % N₂, 10 % H₂, and 10 % CO₂). The bottles were sealed with gas-tight butyl rubber stoppers and secured with aluminum foil caps, and then were transferred to an incubator for fermentation at 35 °C, which was approximately the top temperature during the traditional CSFB fermentation (Hu et al., 2021), and was within the optimal growth range for many known CPBs (Gu et al., 2021; Wang et al., 2021, 2022b; Dai et al., 2024). After ∼3 days, when the butyl stoppers of anaerobic bottles bulge outward due to microbial growth and related gas production, the fermentation was terminated.

The above fermented broth was mixed well and used as seed for 1:10 inoculation for three independent runs of consecutive scale-up fermentations. In total, four consecutive scale-up fermentations (2.5 L, 25 L, 200 L, 2 t) were conducted for CPBs-rich consortia preparation (Fig. 1A). The medium for CPBs-rich consortia preparation was the same as the above, except that the dilution factor for unsterilized fresh huangshui was changed from 2 times to 4 times (2.5 L – 2 t, NNHS2), and additional calcium carbonate (0.5 g/L) and KH_2_PO_4_ (0.5 g/L) were added to buffer pH and supplement phosphorus source, respectively. The fermentation time for every level was about 3 to 4 days, ending upon vigorous gas production. Stages 1–4 of the scale-up fermentation were used to verify that natural huangshui-based medium could successfully enrich CPBs. The above replicates of fresh huangshui (HS), co-fermented huangshui (FHS), and fermented CPB-rich consortia samples were each mixed well and collected to detect their prokaryotic community composition and physicochemical properties. Specifically, for the three samples CC1–CC3, two technical replicates were taken from each biological replicate.

### 2.2 Pre-cultivation of CPBs with new bottom pitmud and five batches of CSFB fermentation

The enriched liquid CPBs-rich consortia from CC4 were used for pre-cultivation with bottom pitmud of three new pits (N1-N3, ≤ 4 years old) (Fig. 1B) in August. Firstly, the CC4 was mixed well with fresh huangshui at a ratio of 4:1 to supplement the nutrition of the medium. Then, the pH was adjusted to 6.5 with Na_2_CO_3_ solution, forming refreshed CPBs-rich consortia, which was then mixed well with the bottom pitmud (depth: 0–8 cm). The mixed bottom pitmud with a final moisture content of approximately 45 % was piled up for anaerobic pre-cultivation. Ultimately, the three pitmud piles underwent a 30–day period of anaerobic stacking fermentation under natural 25–35℃, each with a layer of cover on top to seal off the air. During the cultivation, the culturing pitmud were re-mixed well every 10 days, and additional refreshed CPBs-rich consortia was added to restore the moisture to approximately 45 %, providing nutrients at the same time. Triplicate samples of the above pre-culturing pitmud (CPM_0d, CPM_10d, CPM_20d, and CPM_30d) (three technical replicates each) were collected to ensure statistically robust measurements, including instant moisture detection and subsequent evaluation of prokaryotic communities and physicochemical properties.

Eventually, the above 30–day cultured artificial bottom pitmud were spread evenly for further five consecutive batches of CSFB fermentation. Each batch lasted for 60 days except for the 5th batch in summer, which lasted for approximately 120 days due to the seasonal reasons inherent in the tradition of CSFB fermentation (Cheng et al., 2023). After each batch, the 4-layer pitmud samples (0–1 cm, 1–3 cm, 3–5 cm, and 5–7 cm) at the center and corner sites were collected in accordance with an established sampling method (Zhang et al., 2020). The sampling depth was adopted because there was no significant difference in the new and aged pitmud samples collected at depths of 3–5 and 5–7 cm, thereby guaranteeing sufficient sampling. Regarding the sample names, taking 4-layer bottom pitmud samples of the 1st-batch (B1) at the center site as examples, they were marked as: B1_Cen_01, B1_Cen_13, B1_Cen_35, and B1_Cen_57, respectively (Fig. 1B). Meanwhile, corresponding multi-dimensional samples were collected from new (N) pits (≤ 4 years old) and aged old (O) pits (≥ 40 years old), serving as negative and positive controls respectively. Triplicate samples were collected from most batches (including B1, B2, B5, N, and O), but not the 3rd (B3) and 4th (B4), as only two replicates were obtained due to time constraints during sampling.

### 2.3 Physicochemical analyses

The fresh huangshui (HS), fermented HS (FHS), scale-up fermented CC1–CC4 samples, solid pre-cultured CPM samples (CPM_0d, CPM_10d, CPM_20d, and CPM_30d), and multi-dimensional bottom pitmud samples from five batches of CSFB fermentation were assessed. The three parameter groups were measured using established methods (Zhang et al., 2020), including (1) pH value—a key factor affecting CPB growth (Zhang et al., 2025), (2) potential carbon sources for various CPBs (lactic acid, glucose, ethanol), and (3) metabolites of the caproic acid-producing pathway (acetic acid, butyric acid, caproic acid). Propionic acid and valeric acid were also analyzed to rule out the possible synthesis of odd-chain carboxylic acid by potential CPBs, such as *Megasphaera* (Kim et al., 2019). The ratio of physicochemical properties (except for pH) to time (in days) was calculated to determine and compare physicochemical changes over time during liquid enrichment of CPBs (CC1–CC4) and during solid-state pre-cultivation (CPM_0d–CPM_30d).

### 2.4 Sequencing of 16S rRNA gene amplicons and data analysis

The above samples were used for further analysis of prokaryotic communities by sequencing of 16S rRNA gene amplicons. The meta-DNA was extracted using E.Z.N.A soil kit (D5625–01, Omega, US), according to the manufacturer’s protocols. Then, the V4 region of the 16S rRNA gene was amplified using the universal primer pair 520F (5′-AYTGGGYDTAAAGNG-3′) and 802R (5′-TACNVGGGTATCTAATCC-3′), which targets both bacteria and archaea. Amplicon quality assessment and high-throughput sequencing were conducted by Shanghai Personal Biotechnology Co., Ltd. (Shanghai, China). After sequencing, the raw data was processed through the QIIME2 (version 2022.11) pipeline to obtain high-quality sequences, including steps such as primer removal, adapter trimming, and detection and removal of chimeras, etc. The high-quality sequences were clustered into amplicon sequence variants (ASVs) at 100 % identity. The ASV sequences were annotated using the Silva database (version 138), which is known to classify the 16S rRNA gene sequences of known CPBs at the genus level as *Caproiciproducens* (Zhang et al., 2025). An ASV table with the same sequencing depth was generated for the analysis of prokaryotic composition, the α- and β-diversity. Additionally, based on the annotation results against the Silva (v138), the 19 known CSFB-sourced CPBs (File S1) was used to further validate and re-annotate the taxonomy of the identified CPBs (Table S1). Specifically, the taxonomic assignment was revised from *Caproiciproducens* to the correct corresponding nomenclature of the CPBs (at both genus and species levels) only when the ASV sequences showed 100 % identity. All other ASVs retained their original annotations by Silva (v138).

### 2.5 Statistical analysis

ASV level α-diversity indices were calculated in QIIME2 (version 2022.11). Beta diversity analysis was performed using weighted unifrac distances metrics, visualized via nonmetric multidimensional scaling (NMDS) in R. Potential functions of prokaryotic consortia in bottom pitmud samples were predicted by PICRUSt2 (Phylogenetic Investigation of Communities by Reconstruction of Unobserved States) according to Metabolic Pathways From all Domains of Life (MetaCyc) (Douglas et al., 2019). The predicted reads per million functional units of enzyme genes involved in the synthesis of caproic acid were calculated (Fig. S1). Pearson’s correlation and related significant analyses were further conducted in R with “cor.test ()”, analyzing the correlations among dominant genera (> 5 %) themselves, and between dominant genera and physicochemical properties. The heatmap was generated in R with package “heatmap()”. For the samples from liquid-enrichment or solid-cultivation of CPBs, inter-group differences were assessed using one-way analysis of variance (ANOVA), followed by Tukey’s HSD post hoc test when the ANOVA results were significant. The significance of the difference in relative abundance between *Caproiciproducens* and the most dominant *Caproicibacterium lactatifermentans* LBM19010 in the surface pitmud was assessed using ANOVA. For multidimensional pit-mud samples collected during five batches of CSFB fermentation, a linear mixed-effects model was used to analyze parameter differences. Sampling pit (pit ID) was treated as a random factor to account for inter-pit variability; sampling position (Center vs Corner), sampling depth (0–1 cm, 1–3 cm, 3–5 cm, and 5–7 cm) and fermentation batch (B1–B5) were included as fixed factors. Negative/positive controls (batch = 0) were incorporated into the “batch” factor to compare experimental pitmud with pre-inoculation (negative control) and aged pitmud (positive control). All parameters were modelled as dependent variables. Pairwise comparisons of marginal means were performed with Tukey. All of the above statistical analyses were conducted using SPSS (version 26.0, IBM, USA).

### 2.6 Accession numbers

The raw sequences of all samples (File S2) were submitted to the Sequence Read Archive of the National Center for Biotechnology Information under BioProject SUB15108826 (BioSample accession numbers SAMN46992062 ∼ SAMN46992273).

## 3 Results and discussion

### 3.1 Liquid enrichment of CPBs

**Fig. 2:**
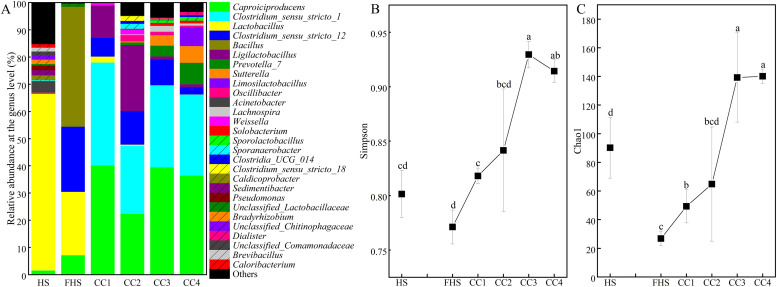
The succession pattern of prokaryotic community structures (A), the variation of the α-diversity parameters Simpson (B) and Chao1 (C) during the preparation of CPBs-rich consortia. Error bars represent standard deviation. Different lowercase letters above bars indicate statistically significant differences, P < 0.05. HS: in-situ Huangshui (HS) as the control; FHS: fermented Huangshui; CC1 ∼ CC4: fermented CPB-rich consortia (CC) of stages 1 ∼ 4. Detailed *p*-values are provided in File S4.

The ASV table obtained from 16S rRNA gene amplicon high-throughput sequencing of samples during CPBs enrichment was shown in File S3. *Caproiciproducens* and *Clostridium_sensu_stricto_12* were the affiliations of known CPBs in Silva (v138) (Zhao et al., 2022; Harahap et al., 2024; Zhang et al., 2025). After pitmud-huangshui co-fermentation, the relative abundance of *Caproiciproducens* increased from 1.46 % in HS to 7.04 % in FHS; meanwhile, the relative abundance of *Clostridium_sensu_stricto_12* increased from 0.04 % (0 ∼ 0.12 %) to 23.94 % (Fig. 2A), indicating the successful enrichment of CPBs. Additionally, the relative abundance of *Lactobacillus* averagely decreased from 64.93 % to 23.38 %. The dominant *Bacillus* (44.08 ± 3.13 %) in FHS was speculated to originate from spores in the pitmud (Xia et al., 2024), which likely proliferated under favorable growth conditions. The relative abundance of *Caproiciproducens* further increased to an average of 34.55 % among the later four levels of successive scale-up culturing (CC1–CC4) (Fig. 2A). Meanwhile, the relative abundance of *Clostridium_sensu_stricto_12* decreased to average 8.65 % in CC1–CC4, while that of *Clostridium_sensu_stricto_1* increased from 0.02 % in FHS to an average of 30.80 % in CC1–CC4. Whereas, the relative abundance of *Lactobacillus* further decreased to 0.70 % (0.01 %–4.31 %) in CC1–CC4, implying that *Lactobacillus* was less competitive than CPBs in the medium. Aerobic or facultative anaerobic *Bacillus* almost disappeared (0.03 %). The above suggested that the relative abundance of CPBs was significantly enhanced during the consecutive liquid batch fermentation from FHS to CC4. Moreover, the abundance (Chao 1) and diversity (Simpson) of the overall prokaryotic community structure reached the maximum at the 3rd and 4th levels of scale-up cultures (Fig. 2B, 2C; File S4). Analyzing the reasons, first, the NNHS medium provided suitable physicochemical conditions for the growth of certain CPBs such as *Clostriduim* (Liu et al., 2014; Wang et al., 2018; Xu et al., 2019) and *Caproicibacterium* (Gu et al., 2021; Wang et al., 2022b; Zeng et al., 2024); second, the NNHS medium was rich in carbon sources (e.g. lactate), which are necessary for the growth of CPBs, as well as nitrogen sources (e.g. NH_4_^+^-N), and precursors (e.g. acetic acid and butyric acid) (Gao et al., 2023; Zhang et al., 2024), providing substrates for the growth of potential CPBs (Table S2); third, the non-sterilized huangshui probably both provided a source of CPBs and growth factors for CPBs (Gao et al., 2020, 2023). Eventually, the originally dominant acidophilic genus *Lactobacillus* in huangshui relinquished its dominance to CPBs (Rault et al., 2009). Further re-annotation of potentical CPBs using identified CPBs using Blastn showed that 65.52 % of *Caproiciproducens* was re-annotated as *Caproicibacterium* (File S5), of which, the most abundant ASV (45.82 % of *Caproiciproducens*) showed 100 % identity with *Caproicibacterium argilliputei* ZCY20–5 (File S6) (Zeng et al., 2024). And, the most abundant ASV of “*Clostridium_sensu_stricto_12*″ and “*Clostridium_sensu_stricto_1*” were 100 %-identity with type strain *Clostridium tyrobutyricum* ATCC 25,755 (Accession: NR_044718.2) and *Clostridium butyricum* ATCC 19,398 (Accession: NR_180,831.1), respectively. Both were butyric acid-producing bacteria (BPBs) (Liu et al., 2006; Chen et al., 2020), consist with the results found by Cao (Cao et al., 2023) and Qiu (Qiu e al., 2024). Therefore, the dominant CPBs and BPBs co-existed in the CPBs-rich consortia, implying a cooperative relationship between them, as verified by Gao (Gao et al., 2022a) and Zhao (Zhao et al., 2024). Additionally, certain amounts of *Sedimentibacter, Weissella, Bacillus*, and other commonly found genera in pitmud were also enriched.

### 3.2 Single-dimensional growth patterns of inoculated CPBs in solid new bottom pitmud

**Fig. 3:**
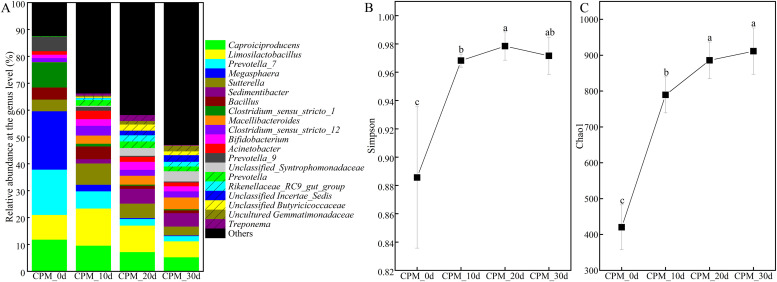
The succession pattern of prokaryotic community structures (A), the variation of the α-diversity parameters Simpson (B) and Chao1 (C) during the solid PM pre-culturing. Error bars represent standard deviation. Different lowercase letters above bars indicate statistically significant differences, P < 0.05. CPM_0d ∼ CPM_30d: solid pre-cultured pitmud samples at 10-day intervals (0, 10, 20, and 30 days). Detailed *p*-values are provided in File S7.

The liquid CPBs-rich consortia were anaerobically cultured with solid new bottom pitmud to track changes of CPBs. At the beginning (CPB_0d, Fig. 3A), the relative abundance of *Caproiciproducens* and *Clostridium_sensu_stricto* were 11.80 % and 10.87 %, respectively, which were significantly lower than in CC4. Then, after 30-day solid-state fermentation, the relative abundance of *Caproiciproducens* gradually decreased to 5.24 %, while the relative abundance of *Clostridium_sensu_stricto* decreased even lower to 2.71 %. *Caproiciproducens* gradually outcompeted *Clostridium_sensu_stricto* in solid-state pitmud, which may be related to its better adaptation to a lactate-dominated substrate environment. Many currently discovered *Caproiciproducens* can degrade lactate and/or glucose (Gu et al., 2021; Wang et al., 2022b, 2022C; Dai et al., 2024; Zeng et al., 2024), while some *Clostridium* primarily metabolize glucose or ethanol as substrates (Liu et al., 2014; Hu et al., 2015). Moreover, compared to liquid systems, the relatively harsher conditions caused CPBs to not maintain the same growth advantage in solid bottom pitmud as they did in liquid systems. Meanwhile, coexisting genera also showed a gradually decreasing trend, such as *Megasphaera*. The genus *Megasphaera* showed competitive advantage at the beginning (21.70 %) and quickly decreased to 2.45 %–0.26 % after 10–day fermentation, which was a potential CPBs that can use lactate for the synthesis of C4–C8 fatty acids, including caproic acid (Fernández-Blanco et a., 2023). Whereas, the relative abundance of others (unidentified or unknown) gradually increased, accompanied by some uncommon genera, such as *Sutterella*, unclassified *Incertae_sedis*, unclassified *Butyricicoccaceae*, and unclassified *Gemmatimonadaceae*. They might have originated from pitmud or the open environment during pitmud mixing. For example, unclassified *Gemmatimonadaceae* are common in soil (Mujakić et al., 2023), deep bottom pitmud. Besides, the pitmud-origin *Sedimentibacter* (4.99 %), *Bacillus* (1.07 %), *Limosilactobacillus*(5.93 %) (originally known as *Lactobacillus* (Zheng et al., 2020)), and unclassified *Syntrophomonadaceae* (3.83 ± 1.11 %) also became dominant in cultured bottom pitmud (Hu et al., 2016; Zhang et al., 2020). Overall, *Caproiciproducens* was the most abundant, which was the characteristic of the prokaryotic community structure of surface bottom pitmud of old aged pit (Zhang et al., 2020). Specifically, after re-annotation with the identified CPBs (Table S1, File S1), 67.06 % of *Caproiciproducens* showed 100 % identity with known *Caproicibacterium* (File S5, File S6). And, the sequence of the most abundant ASV (39.93 % of *Caproiciproducens*) showed 100 % similarity with *Caproicibacterium lactatifermentans* JNU-WLY1368 (File S5) (Wang et al., 2022b). The shift of dominant CPBs from *Caproicibacterium argilliputei* ZCY20–5 in liquid-state CC1–CC4 (Fig.2) to *Caproicibacterium lactatifermentans* JNU-WLY1368 in solid-state bottom pitmud (Fig.3), indicating the adaptive differences of the above two CPBs. Moreover, the abundance (Chao 1) and diversity (Simpson) of the overall prokaryotic community gradually increased and gradually stabilized (Fig. 3B, C; File S7), indicating the steady growing trend of prokaryotic microbiota in new pitmud. In summary, compared to liquid-state fermentation (Fig.2), the coexistence of a lower abundance of CPBs and a higher abundance (Chao1) of the overall microbiota may be related to the complex physical properties of solid-state fermentation (Zhang et al., 2017).

### 3.3 Differences in physicochemical properties between liquid enrichment and solid pre-cultivation

**Fig. 4:**
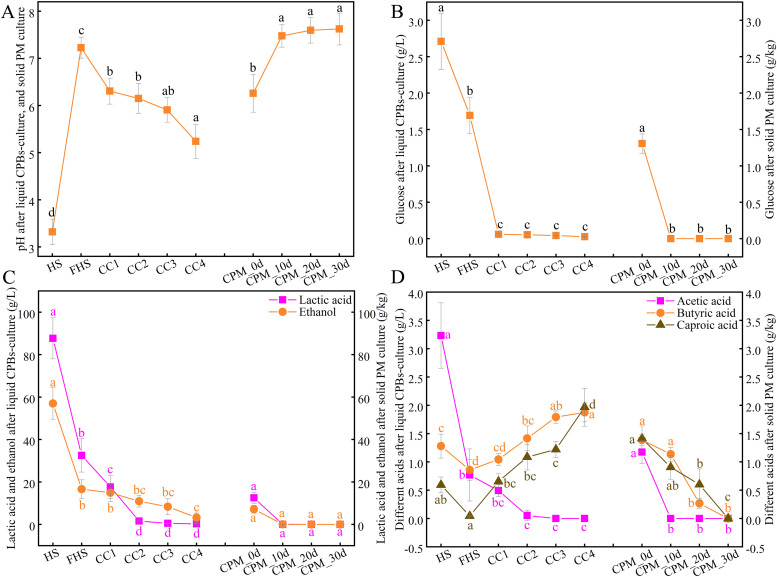
The succession pattern of physicochemical parameters including potential carbon source glucose (A), ethanol, lactic acid (B), caproic acid synthesis metabolites acetic acid, butyric acid, and caproic acid (C), and pH values (D) during the preparation of CPBs-rich consortia and solid PM pre-culturing. Error bars represent standard deviation. Different lowercase letters above bars indicate statistically significant differences within two groups: (HS, FHS, CC1, CC2, CC3, CC4) and (CPM_0d, CPM_10d, CPM_20d, CPM_30d), P < 0.05. Detailed *p*-values are provided in File S8. HS: in-situ Huangshui (HS) as the control; FHS: fermented Huangshui; CC1 ∼ CC4: fermented CPB-rich consortia (CC) of stages 1 ∼ 4; CPM_0d ∼ CPM_30d: solid pre-cultured pit mud samples at 10-day intervals (0, 10, 20, and 30 days).

The differences in physicochemical properties during liquid-state and solid-state fermentations were explored (Fig.4; File S8). First, carbon sources (i.e., glucose, lactate, and ethanol) exhibited a similar trend of gradual reduction during both liquid and solid-state fermentation. Specifically, during the liquid-state scale-up fermentation, the quick carbon source glucose in the medium (Table S2) was depleted after the 1st fermentation (CC1) (Fig. 4A), while lactic acid was depleted after the 2nd fermentation (CC2), and ethanol was exhausted after the 4th fermentation (CC4) (Fig. 4B), indicating that the enriched consortia preferentially utilized glucose, followed by lactic acid, and lastly ethanol. For solid-state fermentation, all three carbon sources were depleted by day 10, which might be related to longer fermentation time and lower carbon source content in the solid bottom pitmud. Notably, most pitmud-sourced CPBs of the *Oscillospiraceae* family can utilize glucose and lactic acid as carbon sources (Wang et al., 2021; Gu et al., 2021, 2022b; Wang et al., 2022c; Zeng et al., 2024; Dai et al., 2024; Jin et al., 2024); and some pitmud-sourced CPBs of the *Clostridiaceae* family can utilize ethanol as carbon sources (Hu et al., 2015). Second, the (intermediate) products of caproic acid synthesis (i.e., acetic acid, butyric acid and caproic acid) exhibited significantly different trends between liquid-state and solid-state fermentations (Fig. 4C). Specifically, during the CPBs enrichment in liquid-state fermentation, acetic acid gradually decreased, while butyric acid and caproic acid gradually increased up to 1.88 g/L and 1.97 g/L, respectively, after the 4th fermentation, with butyric acid increasing earlier, indicating that the potential enriched CPBs in consortia sequentially synthesized butyric acid and caproic acid using acetic acid as the electron acceptor/substrate. However, during solid-state fermentation, acetic acid was rapidly depleted after 10 days, whereas butyric acid and caproic acid were gradually depleted after 30 days, suggesting that the enriched consortia primarily utilized the limited carbon sources for growth rather than for synthesis of caproic acid (Fig. S3). The known reversible RBO carbon chain elongation reaction may also lead to the degradation of caproic acid and butyric acid (Liu et al., 2024). Ultimately, pH decreased during liquid-state fermentation but increased during solid-state fermentation (Fig. 4D). The decreasing pH in liquid-state fermentation may be related to the degradation of added glucose by CPBs and the increase in butyric acid and caproic acid. It is known that the degradation of glucose by CPBs may decrease the pH (Wang et al., 2021). The increasing pH in solid-state pitmud may be related to the degradation of lactic acid and the absence of butyric acid and caproic acid. It is known that the degradation of lactic acid in pitmud leads to an increase in pH (Hu et al., 2016; Zhang et al., 2020; Liu et al., 2022; Wang et al., 2021). In summary, the relative nutritional deficiency of the solid-state system led CPBs to prioritize growth over caproic acid synthesis.

### 3.4 The succession pattern of multi-dimensional bottom pitmud prokaryotic communities during five consecutive batches of fermentation

**Fig. 5:**
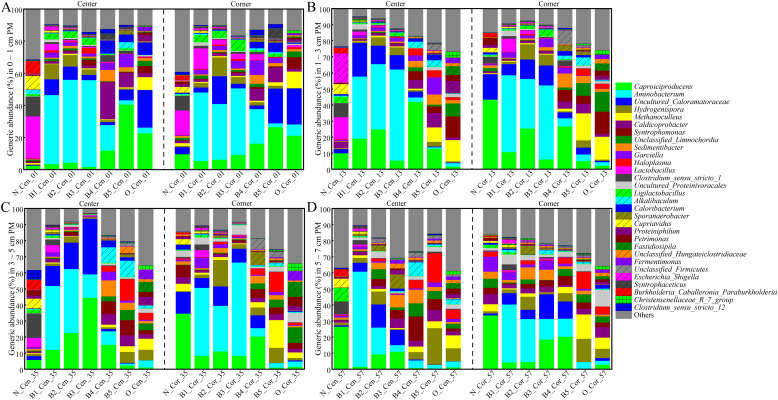
The succession pattern of bottom pitmud prokaryotic community structures at depth of 0–1 cm (A), 1–3 cm (B), 3–5 cm (C), and 5–7 cm (D) from the center (left) and corner (right) during five consecutive batch fermentations. Samples (e.g.): N_Cen_01: new (N) bottom pitmud (0–1 cm, center) as negative control; B1_Cen_01: first-batch fermented bottom pitmud (0–1 cm, center); O_Cen_01: old (O) bottom pitmud (0–1 cm, center) as positive control; B5_Cor_57: fifth-batch fermented bottom pitmud (5–7 cm, corner).

An ASV table obtained by high-throughput sequencing of 16S rRNA gene amplicons of multi-dimensional bottom pitmud samples is shown in File S9. The variation pattern of the prokaryotic communities occurred as follows: (1) *Caproiciproducens* was the most abundant in the 0–1 cm surface layer of bottom pitmud, and the *Caproiciproducens* in the 0–1 cm surface layer of bottom pitmud at the center point (B_Cen_01) showed a slight decrease (3.37 % to 1.72 %) first (B1_Cen_01 ∼ B3_Cen_01); and then (B4_Cen_01 ∼ B5_Cen_01) rapidly re-increased (11.84 % → 40.57 %), approaching the levels of aged pitmud (O_Cen_01) (Fig. 5A); (2) *Caproiciproducens* in the 0–1 cm surface layer of bottom pitmud at the corner point (B_Cor_01) showed a similar pattern, except that the re-increasing phase (B3_Cor_01 ∼ B5_Cor_01) occurred one batch earlier; (3) as compared to the 0–1 cm surface layer of bottom pitmud, *Caproiciproducens* at depth of 1–3 cm (B_Cen_13, B_Cor_13), 3–5 cm (B_Cen_35, B_Cor_35), and 5–7 cm (B_Cen_57, B_Cor_57) showed an opposite trend of first increasing and then decreasing (Fig. 5B, C, D). These results indicated that although originally uniformly distributed, *Caproiciproducens* gradually exhibited a multi-dimensional variation pattern during CSFB fermentation, ultimately distributed in the surface layer at 0–1 cm, which was consistent with our previous study (Zhang et al., 2020). The initial decrease in the abundance of *Caproiciproducens* in artificial pitmud, followed by a gradual increasing trend was consistent with the findings of previous studies (Liu et al., 2020, 2022). Meanwhile, the relative abundance of *Lactobacillus* quickly decreased, and the decreasing rate was faster in the deep than that in the surface layer. The co-occurrence of *Lactobacillus* - decreasing and *Caproiciproducens* – increasing precisely indicated the aging of pitmud (Zhang et al., 2020). In addition, *Aminobacterium* was initially dominant (B1 to B3) and then rapidly decreased (B4 to B5), suggesting that the abundant proteins in huangshui and the slightly neutral pH in the bottom pitmud promoted the proliferation of amino acid fermenting *Aminobacterium* (Baena et al., 1998). However, *Aminobacterium* gradually lost this advantage and maintained its relative abundance within the normal range of old bottom pitmud (Hu et al., 2016; Zhang et al., 2020). Meanwhile, *Caproiciproducens* and its synergistic genera gradually became dominant, such as *Methanoculleus*. The gradual increasing dominance of *Methanoculleus* was consistent with the pattern associated with the aging process of pitmud (Hu et al., 2016; Zhang et al., 2020; Chai et al., 2021; Chen et al., 2024b), although there were some differences in the genera of methanogenic bacteria. Eventually, the dominant genera in the well-recognized aged pitmud became co-dominant after five batches of fermentation, including *Caproiciproducens, Synthrophomonas, Sedimentibacter, Caldicoprobacter, Hydrogenispora, Methanoculleus*, and so on (Tao et al., 2014; Zhang et al., 2020; Hou et al., 2022; Ren et al., 2025; Li et al., 2025). However, the reason why *Caproiciproducens* outcompeted *Clostridium* in the aged pitmud warrants further investigation through isolation of dominant strains and substrate preference assays in future studies.

### 3.5 Succession pattern of α-diversity indices of multi-dimensional bottom pitmud during five consecutive batches of fermentation

Linear mixed-effects model analysis showed that the Simpson index (prokaryotic diversity) differed significantly across fermentation batches (F_4_,_24_ = 7.78, p < 0.001) and depths (F₃,_90_ = 3.61, p = 0.016), whereas the Chao1 index showed no significant differences across batches or depths (F_4_,_6.4_ = 0.91, p = 0.51; F₃,₁_3.7_ = 1.07, p = 0.39), indicating that the prokaryotic communities primarily exhibit succession pattern in diversity across different depths (File S10). However, when the combined effect of “Batch” × “Depth” was tested, the Chao1 index also showed significant variation (F_12_,₁_4.2_ = 3.15, p = 0.021), indicating that the influence of fermentation batch on prokaryotic abundance depends on vertical depth; nevertheless, this interaction effect is relatively weaker than the pattern observed in microbial diversity (Simpson index).

**Fig. 6:**
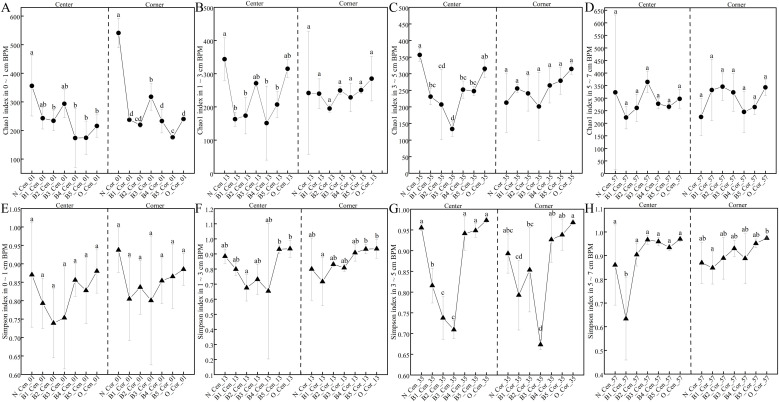
The succession patterns of α-diversity parameters (Chao1 in A-D; Simpson in E-H) for bottom pitmud prokaryotic communities at depths of 0–1 cm (A, E), 1–3 cm (B, F), 3–5 cm (C, G), and 5–7 cm (D, H) from the center (left) and corner (right) during five consecutive batch fermentations. Error bars represent standard deviation. Different lowercase letters above bars indicate statistically significant differences, P < 0.05. Detailed *p*-values are provided in File S11.

The succession trajectories of α-diversity indices at identical depths across successive fermentation batches constituted the major focus of this study. Overall, within-group comparisons of same-depth, different-batch fermentations exerted stronger effects on prokaryotic abundance (Chao1) and diversity (Simpson) in the center bottom pitmud (6/8, excluding only Chao1_Cen_57 and Simpson_Cen_01) than in the corner positions (4/8) (File S11). Specifically, the prokaryotic diversity (Simpson index) initially decreased/stagnated and then increased (Fig. 6, File S11), mirroring the succession pattern of the overall prokaryotic community structure (Fig. 5E∼ H). The decrease/stagnation of the Simpson index corresponded with the decreased abundance of *Caproiciproducens* from B1 to B3, and the increase of Simpson index corresponded with the increase of *Caproiciproducens* from B4 to B5 (Figs. 5, 6). The two distinct phases (B1 to B3 and B4 to B5) showed differences in the Simpson index across different depths. For the bottom pitmud at 0–5 cm, the decrease/stagnation phase was generally from B1 to B3, followed by a re-increasing phase from B4 to B5; whereas for the bottom pitmud at 5–7 cm, the re-increasing phase occurred one batch earlier, or even lost the distinct decrease/stagnation phase. The variation in prokaryotic community abundance (as indicated by the Chao1 index) also roughly demarcated at the B3 irrigation stage (Fig. 5A–D). The Chao1 index tended to increase from B1 to B3 and from B4 to B5, with the exception of an exceptional decrease from B1 to B3 at 3–5 cm. Combining the succession pattern of the prokaryotic community (Fig. 5), distinct differences were observed between the two stages. From the B1 to B3 stage, while prokaryotic diversity (Simpson) decreased, the prokaryotic abundance (Chao1) increased, and the number of prokaryotic genera continued to rise, such as *Aminobacterium*. From the B4 to B5 stage, both prokaryotic diversity (Simpson) and abundance (Chao1) increased along with the abundance of some prokaryotic genera, such as *Caproiciproducens, Synthrophomonas, Sedimentibacter, Caldicoprobacter, Hydrogenispora*, and *Methanoculleus* (Fig. 5). The differences between the two phases, between surface and deep bottom pitmud, as well as between center and corner bottom pitmud, were likely related to the differential responses of pitmud microbiota to the infiltrated huangshui. The microbiota residing in the surface bottom pitmud, acting as the first barrier to the downward infiltration of huangshui, were the primary consumers of the components of huangshui. Therefore, during the first phase, the microbiota in surface bottom pitmud (mainly CPBs) needed more time for adaptation and degradation of the main components of huangshui (e.g. lactic acid), simultaneously producing some products (i.e., acetic acid, butyric acid, and caproic acid) and altering physicochemical parameters (e.g. pH). Neutral or slightly acidic conditions are more conducive to the growth of *Caproiciproducens* (Deng et al., 2024; Zhang et al., 2025). The microbiota residing in the deep bottom pitmud, as the secondary-level consumers of huangshui, faced a lower volume of initially degraded huangshui, and hence less time was needed during the first phase, and further promoted the evolution of microbiota residing in the deep pitmud, which further utilized the degraded products of huangshui by the microbiota of the surface bottom pitmud. For example, hydrogen gas, as a product of caproic acid synthesis, could be used as a substrate for the hydrogenotrophic methanogens (e.g., *Methanoculleus*) in deep pitmud (Lu et al., 2021).

### 3.6 Succession pattern of β-diversity of multi-dimensional bottom pitmud during five consecutive batches of fermentation

**Fig. 7:**
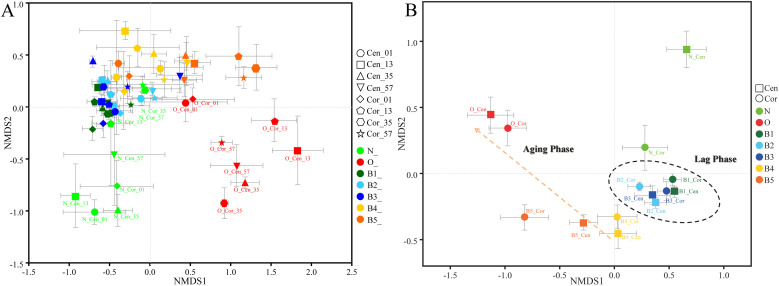
The non-metric multidimensional scaling (NMDS) cluster analysis of multi-layer bottom pitmud prokaryotic communities (A) and integrated groups for each sampling site (B) with the new (N) and matured old (O) pitmud serving as negative and positive controls. The integrated samples are indicated by the mean principal coordinate (PC) score of all samples within each group, and the error bars are indicated by the standard error of the mean. Samples are differentiated by both shape (representing different points and layers) and color (representing different fermentation batches). Integrated samples (e.g.): “O_Cen_01″ group includes replicates of all old bottom pitmud samples (0–1 cm layer, center); “B1_Cor” group includes replicates of all four-layer bottom pitmud samples (corner) after first-batch fermentation.

The results of NMDS analysis revealed distinct clustering patterns: prokaryotic communities from aged bottom pitmud grouped on the right, those from new bottom pitmud on the left, and the five batches of multi-layer bottom pitmud were in the central region (Fig. 7A). This clustering patten was consistent with differences in community compositions (Fig. 5). Notably, among the five batches of multi-layer samples, the communities of the first three batches (B1–B3) were more tightly clustered, whereas those of the last two batches (B4–B5) showed stronger affinity with aged pitmud microbiota, consisting with the succession pattern of prokaryotic communities (Fig. 5) and α-diversity indices (Fig. 6). Moreover, NMDS clustering of integrated bottom pitmud prokaryotic communities further clearly revealed two distinctly divided phases: a lag phase and an aging phase (Fig. 7B).

We hypothesized that huangshui, specifically bacteriostatic lactic acid and ethanol within it, exerted growth pressure on microbiota during the lag phase (B1 to B3) (Kang et al., 2022). Meanwhile, the microbiota was also adapting to the surrounding conditions. The CPBs that could degrade lactic acid and ethanol were enriched later. Then, during the aging phase (B4 to B5), the microbiota formed a complete metabolic chain to degrade various components of huangshui. The degradation capability of bottom pitmud microbiota and the infiltrating rate of huangshui gradually reached a balance. Therefore, during the aging phase, the bottom pitmud microbiota gradually became stable and aged. We designate the above hypothesis as “Lag-aging dual-phase” hypothesis. The aging phase was similar to “Restoration process” by Liu et al. (2022). While confirming the Lag-aging dual-phase” hypothesis, these results contrasted with the findings of Zhou et al. (2024), who reported an initial increase in the abundance of *Caproiciproducens*, followed by a decrease. This discrepancy could be attributed to differences in inoculation. Nonetheless, our findings demonstrated that inoculation with CPBs-rich consortia could effectively accelerate pitmud maturation, reducing the traditional aging period from 20–30 years to just 1 year.

### 3.7 Succession pattern of physicochemical properties in multi-dimensional bottom pitmud during five consecutive batches of fermentations

**Fig. 8:**
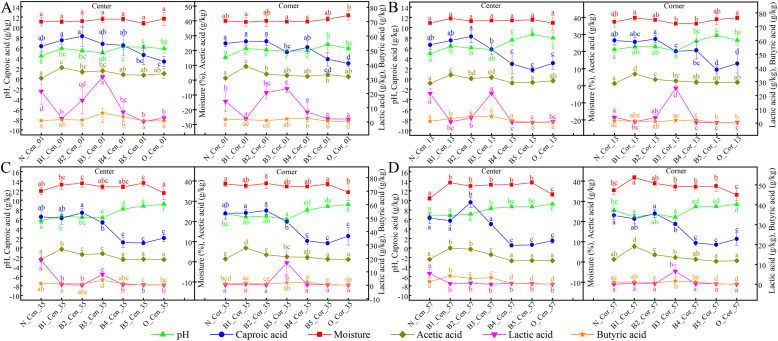
The succession patterns of the physicochemical parameters of pit mud samples at depths of 0–1 cm (A), 1–3 cm (B), 3–5 cm (C), and 5–7 cm (D) from the center (left) and corner (right) during five consecutive batch fermentations. Error bars represent standard deviation. Different lowercase letters above bars indicate statistically significant differences, P < 0.05. Detailed *p*-values are provided in File S13.

The “Lag-aging dual-phase” hypothesis was further verified by the succession pattern of physicochemical properties. Most physicochemical properties, except for water content, showed significant variations across fermentation batches (Batch, p < 0.001) and sampling depths (Depth, p < 0.001); by contrast, the effect of sampling position (Cen vs. Cor) was not significant for certain parameters such as pH (F₁,_74_ = 1.20, p = 0.28) and caproic acid (F₁,_4.78_ = 1.01, p = 0.36), indicating that fermentation batch and vertical depth are the primary drivers of changes in pitmud physicochemical parameters, whereas lateral position exerts a limited influence (File S12). Of course, a significant interaction between “Batch” and “Depth” was also observed, indicating that the impact of fermentation batch on pitmud physicochemical parameters varies across different vertical depths. Given that batch-depth interactions predominantly shaped the physicochemical landscape, we next focused on the detailed succession dynamics of physicochemical properties in multi-depth pitmud over five consecutive fermentation batches (Fig. 8, File S13). From B1 to B3, the physicochemical properties of surface bottom pitmud at 0–1 cm were as follows: (1) lactic acid significantly accumulated; (2) the contents of acetic acid, butyric acid, and caproic acid decreased slightly; and (3) the pH value and moisture content were basically stable. According to the “Lag-aging dual-phase” hypothesis, the “B1 to B3” was the “Lag phase”. The infiltered large amount of huangshui led to the accumulation of the main component, lactic acid, in the surface bottom pitmud, while the weak metabolic level of the inoculated CPBs led to slight synthesis or degradation of acetic acid, butyric acid, and caproic acid. Therefore, the pH and moisture content were relatively stable. As compared to the surface bottom pitmud, the “Lag phase” of the deeper bottom pitmud appeared later, was shorter, and exhibited a smaller amplitude of change to physicochemical property. For example, lactic acid accumulated one batch later, and the accumulation amplitude became smaller or even disappeared (Fig. 8B, C, D), which might be related to the delayed seepage of a smaller amount of huangshui from the surface into the deeper layers. The bottom pitmud was like the fixed “packed bed” of the traditional CSFB fermenter –fermenting pit, which reduced the rate of huangshui seepage from the surface into the deeper bottom pitmud, at least to some extent, where huangshui served as substrate for the microbiota of the bottom pitmud, affecting the metabolism of the microbiota, which then affected the physicochemical properties of the bottom pitmud, thereby further influencing metabolism of the microbial community…

From B4 to B5, the physicochemical properties of the surface bottom pitmud at 0–1 cm were as follows: (1) the amount of lactic acid gradually decreased; (2) the caproic acid content gradually decreased; (3) the acetic acid, butyric acid, and moisture contents were relatively stable; and (4) the pH value gradually increased. According to the “Lag-aging dual-phase” hypothesis, the “B4 to B5” was the “Aging phase”. The inoculated CPBs began to grow, thence the abundance of *Caproiciproducens* gradually increased (Fig.5). The metabolism of CPBs probably degraded lactic acid, leading to the continuous decrease of lactic acid, gradually approaching the levels of the aged bottom pitmud (Fig. 8A). Meanwhile, the degradation of lactic acid promoted a slight increase in the pH values. Compared with the surface bottom pitmud, the “Aging phase” of deeper bottom pitmud showed a similar but smaller amplitude of physicochemical property change, which was potentially attributable to reduced infiltration of huangshui. Notably, the co-occurrence of the degradation of carbon source lactic acid and the increase of *Caproiciproducens* did not promote the synthesis of butyric acid and caproic acid during the “Aging phase”. Instead, the level of caproic acid decreased. This was basically consistent with situation during the solid-state pre-culturing of CPBs-enrich consortia (Fig.4). Hence, it was speculated that the CPBs-enrich consortia primarily utilized the relatively insufficient amount of nutrients for growth rather than caproic acid synthesis. Comparatively, the significantly lower concentrations of propionic acid (0.00–0.23 g/kg) and valeric acid (0.02–0.30 g/kg) further demonstrated the metabolic preference of CPB-rich consortia for caproic acid synthesis (Fig. S2). Compared with liquid-state fermentation with uniform and abundant substrates, the CPBs grew slower in the complex multi-dimensional solid system characterized by non-uniform and limited nutrients (Zhang et al., 2017). Caproic acid synthesis, as the subsequent metabolism of the carbon source metabolite (acetyl-CoA) (Liu et al., 2024), was relatively delayed. In this study, the decreasing trend of butyric acid and caproic acid during the “Aging phase” was consistent with that during the natural aging of pitmud (Tao et al., 2014) and the changes in butyric acid and caproic acid in 3-year, 20-year, and 50-year PMs found by Xia et al. (2024) in the area (104º 40′ 32″ E, 30º 5′ 37” N), which tended to first decrease and then increase. It was speculated that in this study, due to insufficient cultivation time, the growing CPBs had not yet focused on caproic acid synthesis, and so, the contents of butyric acid and caproic acid did not yet show an upward trend.

### 3.8 Succession pattern of pearson’s correlations between *Caproiciproducens* and dominant genera, and between dominant genera and physicochemical properties

**Fig. 9:**
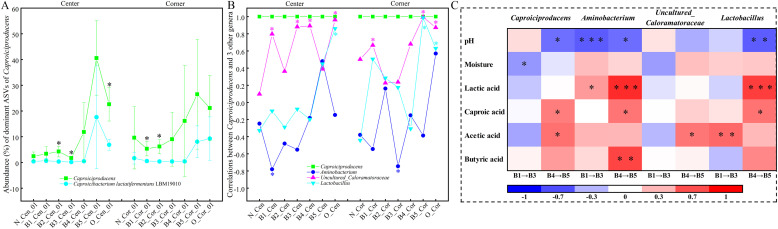
The succession patterns of the relative abundance of *Caproiciproducens* and its dominant ASVs in the pitmud samples of surface layer 0–1 cm (A); the strong Pearson correlation between *Caproiciproducens* and dominant genera (> 3 %) (B); and the Pearson correlation (> 0.5) between dominant genera and physicochemical parameters (C) (significant differences or correlations, *** indicate P < 0. 001, ** indicate P < 0.01, * indicate P < 0.05). Detailed *p*-values are provided in File S16.

Lactate-guided *Caproiciproducens* was recognized as the most abundant and the main force to convert lactic acid into caproic acid (Jin et al., 2024). Therefore, the succession pattern of *Caproiciproducens* and dominant ASVs was first discussed. The abundance of *Caproiciproducens* was relatively low (4.98 %) during the “Lag phase” (B1 to B3), slightly lower than its abundance in CPM_30d (5.24 %, Fig. 3), indicating an actual decrease during the “Lag phase” phase. However, upon entering the “Aging phase” (B4 to B5), the abundance of *Caproiciproducens* rapidly increased, and approached or even exceeded the level of aged pitmud. And, the most dominant identified ASV of *Caproiciproducens* (File S9, File S14, File S15), accounted for 26.21 % and showed 100 % identity with strains *Caproicibacterium lactatifermentans* LBM19010 (Fig. 9A, File S16), consistent with a report by Wang et al. (2021).

Interestingly, *Caproiciproducens* and other dominant genera (> 3 %) showed relatively low Pearson’s correlations during the “Lag phase” (B1 to B3). Then, upon entering the “Aging phase” (B4 to B5), the correlation coefficients rapidly increased, further indicating that during the “Aging phase”, *Caproiciproducens* formed a strong metabolic network with dominant genera, such as *Aminobacterium, Lactobacillus*, and *Uncultured_Caloramatoraceae* (Fig. 9B, File S16). It was speculated that the protein degradation function of the dominant genus *Aminobacterium* (Baena et al., 1998) and the acetic acid synthesis function during degradation of lactic acid by *Lactobacillus* (Özcelik et al., 2016) provided nitrogen sources and substrates for caproic acid synthesis and metabolism by *Caproiciproducens*. This result was consistent with the conclusions of previous studies on the correlation between prokaryotic genera during the evolution of pitmud (Zhou et al., 2023b; Xia et al., 2024).

Interestingly, the Pearson’s correlation between the above dominant genera and the physicochemical parameters (i.e., moisture, lactic acid, acetic acid, butyric acid, and caproic acid) gradually changed from weak or negative during the “Lag phase” (B1 to B3) to strong or positive during the “Aging phase” (B4 to B5). Whereas, Pearson’s correlation between *Caproiciproducens* and the pH value shifted from weakly positive during the “Lag phase” (B1 to B3) to significantly negative during the “Aging phase” (B4 to B5) (Fig. 9C, File S16). The co-occurrence of differences in CPBs abundance, inter-genus relationships, and genus–physicochemical parameters between the two phases indicated significant shifts in microbial metabolic functions and their consequent impacts on the surrounding physicochemical environment. Further studies are warranted to investigate these functional differences.

### 3.9 Functional prediction of caproic acid synthesis in multi-dimensional bottom pitmud during five consecutive batches of fermentation

**Fig. 10:**
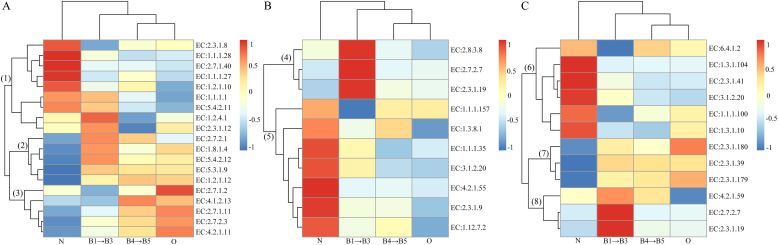
Changes in abundance of functional genes for metabolic enzymes involved in the substrates (include ethanol, Glucose, lactic acid) degradation (A), RBO (reverse β-oxidation cycle) (B), and FAB (fatty acid biosynthesis cycle) (C) caproic acid synthesis pathways predicted by Picrust2. Enzymes related to substrates degradation in Figure 10A: pyruvate decarboxylase (EC:4.1.1.1); alcohol dehydrogenase (EC:1.1.1.1); l-lactate Dehydrogenase (EC:1.1.1.27); d-lactate Dehydrogenase (EC:1.1.1.28); acetaldehyde dehydrogenase (EC:1.2.1.10); pyruvate dehydrogenase (EC:1.2.4.1); glucokinase (EC:2.7.1.2); phosphohexose isomerase (EC:5.3.1.9); 6-phosphofructokinase (EC:2.7.1.11); diphosphofructose aldolase (EC:4.1.2.13); 3-phosphoglyceraldehyde dehydrogenase (EC:1.2.1.12); phosphoglycerate kinase (EC:2.7.2.3); enolase (EC:4.2.1.11); pyruvate kinase (EC:2.7.1.40); PTA, phosphotransacetylase (EC:2.3.1.8); ACK, acetate kinase (EC:2.7.2.1). Enzymes related to RBO in Fig. 10B: THL, acetoacetyl-CoA thiolase (EC:2.3.1.9); KCR, ketoacyl-CoA reductase (EC:1.1.1.35, 3-hydroxyacyl-CoA dehydrogenase; EC:1.1.1.157, 3-hydroxybutyryl-CoA dehydrogenase); HCD, 3-hydroxybutyryl-CoA dehydratase (EC:4.2.1.55); ECR, short-chain acyl-CoA dehydrogenase (EC:1.3.8.1); H2ase, ferredoxin hydrogenase (EC:1.12.7.2); TES, acyl-CoA thioesterase (EC:3.1.2.20); BUK, butyrate kinase (EC:2.7.2.7); PTB, phosphate butyryltransferase (EC:2.3.1.19); BUT, butyryl CoA:acetate CoA transferase (EC:2.8.3.8). Enzymes related to FAB in Fig. 10C: ACC, acetyl-CoA carboxylase (EC:6.4.1.2); ATA, acetyltransacylase; MTA, ACP-malonyltransferase (EC:2.3.1.39); KAS, ketoacyl-ACP synthase (EC:2.3.1.41, beta-ketoacyl-ACP synthase I; EC:2.3.1.179, beta-ketoacyl-ACP synthase II; EC:2.3.1.180, beta-ketoacyl-ACP synthase III); KAR, beta-ketoacyl-ACP reductase (EC:1.1.1.100); HAD, 3-hydroxyacyl-ACP dehydratase; EAR, enoyl-ACP reductase (EC:1.3.1.10).

Although transcriptomic analysis could provide stronger support for functional profiling, the complex physicochemical composition of pitmud presented unique challenges for transcriptome extraction, as comprehensive transcriptomic profiling remained unresolved (Mei et al., 2025). Therefore, based on the accuracy, reproducibility, and gene prediction capability of 16S rRNA gene amplicon sequencing - based PICRUSt2 (Johnson et al., 2019; Sun et al., 2020), the abundance of currently known genes encoding enzymes involved in caproic acid biosynthesis were predicted using PICRUSt2 (Fig. 10), which included carbon-source degradation, RBO and FAB caproic acid biosynthesis pathways (Fig. S1). The abundances of genes predicted to encode carbon-source degradation, RBO and FAB enzymes of the caproic-acid synthesis pathway in the “Aging phase” (B4 to B5) clustered closer to those in aged pit-mud (O), indicating functional convergence in caproic-acid synthesis between the two groups. However, the “Lag phase” (B1 to B3) pitmud samples were more concentrated on lactic acid degradation (cluster (1) of Fig. 10A) and butyric acid synthesis both via RBO (cluster (6) of Fig. 10B) and FAB (cluster (7) of Fig. 10C) pathways, rather than caproic acid synthesis. These predictions were consistent with the conclusion drawn from the prokaryotic community (Fig. 5) and physicochemical property analysis (Fig. 8). It was speculated that the “Lag phase”, as the transition stage from liquid state to the solid-state pitmud, probably underwent significant changes. Further studies are needed to investigate the metabolic mechanisms related to caproic acid synthesis during the “Lag phase”, while shortening the “Lag phase” to promote earlier entry into the “Aging phase”.

## 4 Conclusion

This study demonstrated that inoculation with autochthonous CPB-rich consortia enabled four-year-old pitmud to achieve physicochemical parameters and prokaryotic community structures approximating those of 40-year-old pitmud, thereby accelerating pitmud aging. During CPBs enrichment, changes to the physicochemical properties between the liquid-state and solid-state fermentations of CPBs indicated that in the liquid-state system, CPBs primarily engaged in growth and caproic acid synthesis; whereas, in the solid-state system, growth was the main activity. During the aging process after CPBs inoculation, *Caproiciproducens* were the key in the surface pitmud.

The succession pattern of prokaryotic communities and physicochemical properties in multi-dimensional bottom pitmud during five consecutive batches of fermentations indicated that the aging process of bottom pitmud might involve two distinct phases, the “Lag phase” (“adaptive phase”) from batch 1 to batch 3 (B1 to B3) and the “Aging phase” from batch 4 to batch 5 (B4 to B5). The dominant CPBs *Caproiciproducens* were mainly distributed in the surface layer (0–1 cm) of the bottom pitmud, whose abundance in center point initially slightly decreased during the “Lag phase” and later increased during the “Aging phase”; whereas the “Aging phase” of the corner site occurred one batch earlier (B3 to B5). *Caproiciproducens* in the deeper layers (3–7 cm) of bottom pitmud exhibited an opposite trend of first increasing and then decreasing, accompanied by an increase in synergistic genera, such as *Methanoculleus*.

The proposed “Lag-aging dual-phase” hypothesis was further co-verified through the succession patterns of prokaryotic community α-diversity indices, NMDS β-diversity clustering, Pearson’s correlations among dominant genera themselves, and between dominant genera and physicochemical properties, and the functional prediction of enzymes involved in caproic acid synthesis by PICRUSt2. The “Lag-aging dual-phase” hypothesis proposed in this study could be advanced as a prospective theory to elucidate the key role of CPBs for the aging of bottom pitmud. Moreover, this *in situ*-validated theory provided a solid basis to optimize the quality of bottom pitmud. Although this model successfully established a correlation of *Caproiciproducens* in surface-bottom pitmud with aging of bottom pitmud, the underlying regulatory mechanisms remain unclear. Future work combining metagenomics, transcriptomics and enzyme kinetics could bridge this gap. The method employed in this study to accelerate pitmud aging may allow access to premium CSFB by reducing reliance on scarce aged pitmud resources. Furthermore, huangshui-based CPBs fermentation is of negligible cost.

## Funding

This work was financially supported by the 10.13039/501100001809National Natural Science Foundation of China (NSFC) (Grant 32302031), the Provincial Key R&D Program of Anhui Province (Grant 2022n07020002), and the Opening Project of Anhui Engineering Laboratory for Industrial Microbiology Molecular Breeding (Grant ELMB-05).

## CRediT authorship contribution statement

**Huimin Zhang:** Conceptualization, Methodology, Investigation, Writing – original draft, Formal analysis, Funding acquisition. **Li Zhen:** Investigation, Writing – review & editing, Formal analysis. **Qiang Chang:** Data curation, Project administration, Resources, Investigation. **Yan Liu:** Methodology. **Kangjie Xu:** Data curation. **Xiuben Wang:** Data curation, Methodology, Formal analysis, Software, Visualization. **Lei Cui:** Resources, Validation. **Zaijie Wu:** Investigation, Resources. **Zhenglian Xue:** Resources, Writing – review & editing, Supervision.

## Declaration of competing interest

The authors declare that they have no known competing financial interests or personal relationships that could have appeared to influence the work reported in this paper.

## Data Availability

Data will be made available on request.

## Appendix Supplementary materials

Image, application 1
